# Editorial: Living with and beyond cancer across the lifespan

**DOI:** 10.3389/fpubh.2025.1728118

**Published:** 2025-10-29

**Authors:** Ismail Toygar, Annie Young, Gülcan Baǧcivan, Paz Fernández-Ortega

**Affiliations:** ^1^Fethiye Faculty of Health Sciences, Mugla Sitki Koçman University, Mugla, Türkiye; ^2^Warwick Medical School, University of Warwick, Coventry, United Kingdom; ^3^Department of Adult Nursing, College of Nursing and Health Sciences, University of Massachusetts Dartmouth, North Dartmouth, MA, United States; ^4^Instituto Catalán de Oncología, Universidad de Barcelona, Grupo Consolidado GRIN, IDIBELL, Hospitalet de Llobregat, Barcelona, Spain

**Keywords:** cancer, survivorship, care, oncology, health

In recent decades, the population of individuals living with or beyond cancer has increased, a development largely attributable to advancements in improved awareness and prevention, early diagnosis, new drugs and cell therapies, and more precise surgical and radiotherapy techniques. However, these advancements have not benefited everyone; there are significant inequities in cancer outcomes and attention to living with and beyond cancer worldwide (1). The Global Cancer Observatory dataset, published by the World Health Organization, showed that the global population of cancer survivors is estimated to be 50 million, with 18 million residing in the United States and 32 million residing in other regions of the world (2).

The needs and experiences of this growing population have become increasingly significant in recent decades (3). Those living with and beyond cancer may be affected physically; nonetheless, the psychological and social challenges are often as distressing. These challenges require a holistic and multidisciplinary approach for individuals living with and beyond cancer.

The studies gathered in this Research Topic build on this premise by exploring a wide range of settings, diagnoses, and methodological traditions. For example, Chen, Han et al. analyzed data from the National Health and Nutrition Examination Survey (NHANES) and demonstrated a U-shaped relationship between sleep duration and mortality among cancer survivors. The authors further showed that adequate physical activity can mitigate the risks associated with both short and long sleep. However, they also noted that inadequate physical activity can have a synergistic effect, amplifying the risk at sleep extremes. In their seminal study, Wang et al. examined nasopharyngeal cancer survivors and reported a high prevalence of depression, anxiety, and sleep disorders. The risk of these conditions increased with older age at diagnosis and peaked within months after diagnosis, before easing around 12 months. This emphasizes the need for early psychological support. In Romania, Kállay et al. found that poorer sexual health and a greater fear of progression were associated with depression, anxiety, a younger age, a lower level of education, living in a rural area, and a lower level of satisfaction in communication with clinicians. This underscores a frequently disregarded aspect of survivorship. In their 2024 study, Ahmed et al. found that 58.4% of Palestinian women diagnosed with breast cancer experienced death anxiety. The researchers found that active coping strategies were associated with reduced odds of anxiety, while some forms of religiosity and self-blame were associated with higher odds of anxiety. These findings suggest that there are nuanced, culturally influenced targets for psycho-oncology. Turning to caregivers, Xu et al. conducted a prospective, mixed-methods study of spousal caregivers of young and middle-aged patients with terminal cancer in China and found that spousal social support, the availability of alternative caregivers for other dependents, and the number of venous pathways/instruments on the patient were significant predictors of caregivers' stress responses. Qualitatively, four “death-care” themes emerged: caring for patients' physical function, communicating with patients, anticipatory widowhood, and a shift in life focus and values. Addressing the issue of symptom burden, Asefa et al. highlighted the issue of cancer-related fatigue in Ethiopia, which was found to have a prevalence of 77.4%. The study identified late stage, anemia, comorbidities, and lack of insurance as significant correlates. Interviews revealed that financial strain and symptom burden were key factors contributing to fatigue. Patients' information needs are also clearly expressed: Caprara et al. conducted a study that involved the mapping of nutrition-related inquiries posed by Italian breast cancer survivors (*n* = 1,159). The findings of this study indicated a pronounced demand for evidence-based guidance. In response to this need, Caprara et al. implemented a pilot program comprising Instagram Live sessions and a provider workshop. Moving on to movement-based interventions, a review of Tai Chi/Baduanjin randomized controlled trials (RCTs) in breast cancer was conducted by Chen, Zuo et al., revealing improvements in anxiety, depression, sleep, fatigue, and quality of life. However, the researchers cautioned that heterogeneity and the overall quality of the evidence necessitate more rigorous trials. Furthermore, Xiong et al. synthesized 48 RCTs and identified an optimal exercise “dose“ of ~850 MET-min/week to maximize health-related quality of life, with mixed training ranking highest and diminishing returns observed beyond ~1,100 MET-min/week—an actionable benchmark for survivorship programs.

When considered collectively, these contributions advocate for a comprehensive survivorship care model. This comprehensive model should include early and frequent screening for mental health concerns post-diagnosis, normalization of discourse surrounding sexual wellbeing, the acknowledgment of caregiver stress as a clinical concern, adaptation of care to align with patients' preferred sources of information, and prescription of movement with clearly defined, data-driven targets, while considering culturally appropriate modalities. In summary, “living with and beyond cancer” is not a single phenomenon, but rather a series of interconnected physiological, psychological, and social adjustments. The articles in this Research Topic provide a comprehensive overview of the experiences of individuals living with and beyond cancer. As a global cancer community, we will work together to reach other countries where these programs do not exist.
